# Supplementary material for: Prevalence of violence and associated factors among youth in Northwest Ethiopia: Community-based cross-sectional study
Source: PLoS One. 2022 Aug 2;17(8):e0264687. doi: 10.1371/journal.pone.0264687 (PMC9345350; doi:10.1371/journal.pone.0264687)
Supplement: Attachment — Submitted filename: Response to Reviewers.docx [file pone.0264687.s003.docx]

To: Dear, Siew Ann Cheong, Ph.D
PLOS ONE
Subject: Submitting a revised version of manuscript and point by point response

PONE-D-20-39684
Prevalence of violence and associated factors among Youth in Northwest Ethiopia: Community-based Cross-sectional study

**Point by point response**

| **Response for Editor**  1. When submitting your revision, we need you to address these additional requirements.  Please ensure that your manuscript meets PLOS ONE's style requirements, | Thank You Dear,   - Authors have made corrections on the manuscript by using the PLOS ONE’s style. |
| --- | --- |
| 2. Please provide additional details regarding participant consent. In the ethics statement in the Methods and online submission information. | - This is also corrected in the manuscript |
| 3. Please update your submission to use the PLOS LaTeX template. | - Authors, We have done correction based on this instruction |
| 4. The University of Gondar had sponsored this study. However, it has no role in manuscript preparation and publication." At this time, please address the following queries:  a) Please clarify the sources of funding (financial or material support) for your study. List the grants or organizations that supported your study, including funding received from your institution.  b) State what role the funders took in the study. If the funders had no role in your study, please state: “The funders had no role in study design, data collection and analysis, decision to publish, or preparation of the manuscript.” c) If any authors received a salary from any of your funders, please state which authors and which funders. | a) The University of Gondar supported the staffs financially to conduct this research. b) The funder had no role in this research: had no role in study design, data collection and analysis, decision to publish, or preparation of the manuscript. c) All authors are received their salaries from University of Gondar except Temiro Azanaw Mengistu and Yilikal Tiruneh Ayele. |
| 5. Thank you for stating the following:  a. Review your statements relating to the author contributions, and ensure you have specifically and accurately indicated the role(s) that these authors had in your study. These amendments should be made in the online form.  b. Confirm in your cover letter that you agree with the following statement, and we will change the online submission form on your behalf:  “The funder provided support in the form of salaries for authors [insert relevant initials], but did not have any additional role in the study design, data collection and analysis, decision to publish, or preparation of the manuscript. The specific roles of these authors are articulated in the ‘author contributions’ section. | - "The University of Gondar had sponsored this study. However, it has no role in manuscript preparation and publication."  a) Authors, we have made correction in the main document. b) Corrected. |
| 6. In your Data Availability statement, you have not specified where the minimal data set underlying the results described in your manuscript can be found | - All relevant data are within the manuscript and its Supporting information files. |
| 7. PLOS requires an ORCID iD for the corresponding author in Editorial Manager on papers submitted after December 6th, 2016. Please ensure that you have an ORCID ID and that it is validated in Editorial Manager | Thank You. |
| 8. Your ethics statement should only appear in the Methods section of your manuscript. If your ethics statement is written in any section besides the Methods, please move it to the Methods section and delete it from any other section | - It is moved to the appropriate place. |
| **Reviewers' comments** | |
| **Reviewer #1:**  **Abstract:** Page 2, line 33, the authors stated that “There was limited information on the burden of violence and factors among this age group”. This is not true | - Thank you for providing an important comments and questions. - Even if there were a lot of studies in different setting, those studies mainly concerned on institution based, the study participants were adolescent females or males only, researched a single problem (sexual or physical). Whereas, this study was community based including both sex group, assessed overall prevalence of violence in general and the three common forms of violence in particular. |
| Page 2, lines 39-40, variables/risk factors used in the logistic model should be specified in the abstract. What was the dependent variable? What were the independent variables? | Thank You!   - Youth violence was the dependent variable whereas socio-demographic variables (age, sex, marital status, religion, residence, occupation, educational level, currently attending school, with whom you live, wealth index) and substance use were the independent variables. |
| Page 3, lines 47-48, is it a new sentence for “Living with mother’s only... ”? This part should be checked for typo and reorganized for better understanding. | - It has no any error. When the data collector asked an individual’s living arrangement. The response of the participant was living with mother’s only meaning the respondent was living only with his or her mother. |
| **Introduction:** Page 3, line 63, need a citation for the sentence “It is estimated that each year, 200,000 homicides occur in this age group in the world.”. | Thank you,   - Authors accepted the comments and corrected it accordingly. |
| Pages 4-5, lines 82-108, the authors introduced many violence-related studies involving youth around the world. What was the core information the authors tried to deliver? As we all know, the prevalence of violence has been well studied; in some countries, surveillance data are available to monitor the trend and consequences of youth risk behaviors including violence. The authors may want to reorganize the literature and see how they can better serve their paper. | - The core information was to know how much the magnitude of youth violence in different countries and settings in general and types of violence in particular and also to look-over a limited studies on youth violence in Ethiopia. These reviews used for the authors to compare their own research findings accordingly. |
| Page 6, lines 123-129, what are the gaps in the current literature? The authors need to briefly summarize the findings from pervious violence-related studies that included youth in Ethiopia, and then specify the gaps that this study may bridge. If very limited studies were found, studies in other countries with similar demographic characteristics or socio-economic status should be highlighted to provided information instead. | - The gaps in these limited literatures were the studies mainly concentrated among women age 15 to 49 or only on males, and institutional based. Whereas, this study concerned on both sex, among youth and community based and also assessed the three forms of violence simultaneously. - Studies in other countries with similar demographic characteristics or socio-economic status might have different socio-cultural and behavioral characteristics. There might be also a variation on the associated factors. So, Identifying the prevalence and associated factors of youth violence was helpful to design an intervention in the study area. |
| **Methods:** Page 7, lines 147-149, the authors mentioned: “Firstly, two woredas from the central Gondar zone, one woreda from each North Gondar zone and west Gondar zone were selected. Secondly, three kebeles were selected from the selected woredas.” How were the woredas and kebeles selected? Details need to be provided. | - Centeral Gondar having 13 woredas (north Gondar zone having 7 woredas and west Gondar zone having 4 woredas). - “Firstly, two woredas from the central Gondar zone, one woreda from each North Gondar zone and west Gondar zone were selected by using simple random sampling tecnique. - Secondly, from the total number of kebeles (546 kebeles) in the three zones: three kebeles were selected from each selected woredas by simple randomly sampling. |
| Pages 7-8, lines 154-167, what were the variables/survey questions used in this study? Only definitions from WHO are insufficient for readers to learn this study. The authors may benefit from providing a detailed description of the survey, including how it was developed, included domains/topics, and questions/valid responses used in this study. | The survey questions were   - Have you experienced violence in the past 12 months? - Have you experienced violence like hit /slapped or thrown something by someone in the past 12 months? - Have you experienced violence like scared/intimidated by somebody in the past 12 months? - Have you exposed to sexual violence for the last 12 months? |
| Pages 8-9, lines 180-185, was it a complete dataset for analysis? Any missing data information? | - Yes, No any missing data information |
| **Results:** Pages 9-13, table 1 and table 2 can be combined, as both addressed the participants’ demographic information | Thank You,   - Table 1 and table 2 are merged into one table. |
| Page 13, lines 201-205, it seems there were multiple violence variables. Which one was the dependent variable used for the logistic regression? | - The dependent variable used for the logistic regression was “prevalence of violence” |
| Page 15, line 201, what were the independent variables that tested in the regression? If all of them were listed in table 4, why they were selected? Are there any other variables in the survey that can be potentially taken into the model (e.g., mental health problems)? | - No, all variables listed in the socio-demographic table were tested in the bi-variable analysis. Those variables have a p-value of < 0.2 were taken in to the multivariable logistic regression model. Those variables are listed in the table 4(3) and other potential variable like substance use was considered from the survey. |
| **Discussion:** This section repeated too much of the results (e.g., lines 227, 235, 248, 251...). Also, the authors may want to discuss the strengths of this study and implications for violence prevention | - It has no any repetition; the authors wanted to describe how much the prevalence of different forms of youth violence (physical, sexual or emotional) and compared them with other studies. |
| **Reviewer #2**  There is limited information about assessing risk of bias | Thank you very much!   - In addition to recall bias, social desirability bias might be occurred during data collection since the data collectors were used interviewer administered questionnaires. |
