# Supplementary material for: Prevalence of violence and associated factors among youth in Northwest Ethiopia: Community-based cross-sectional study
Source: PLoS One. 2022 Aug 2;17(8):e0264687. doi: 10.1371/journal.pone.0264687 (PMC9345350; doi:10.1371/journal.pone.0264687)
Supplement: Attachment — Submitted filename: Revised Response to reviewers.docx [file pone.0264687.s004.docx]

To: Dear, Siew Ann Cheong, Ph.D
PLOS ONE
Subject: Submitting a revised version of manuscript, Manuscript with Track Changes and point by point responses

PONE-D-20-39684R1
Prevalence of violence and associated factors among Youth in Northwest Ethiopia: Community-based Cross-sectional study

**Point by point response**

| **Response for Editor** |  |
| --- | --- |
| Please ensure that your manuscript meets PLOS ONE's style requirements, | Dear Editor, we have tried to address the PLOS ONE’s requirements. |
| **Reviewer #1:**  **Abstract:** Page 2, line 33, the authors stated that “There was limited information on the burden of violence and factors among this age group”. This is not true. | Dear reviewer #1, Author’s have accepted your complaint, however, we couldn’t understand which areas/questions were not well addressed.  Anyway, we have made modification on some questions as much as possible.   - Authors reviewed a lot of studies however, those studies mainly concerned on institution based, the study participants were adolescent females or males only, researched a single problem (sexual or physical) and didn’t show burden of violence among this specific age group. Whereas, this study was community based including both sex group, assessed overall prevalence of violence in general and the three common forms of violence in particular. |
| Page 2, lines 39-40, variables/risk factors used in the logistic model should be specified in the abstract. What was the dependent variable? What were the independent variables? | Thank You!   - “Youth violence” was the dependent variable whereas all socio-demographic variables listed in table1 and substance use were the independent variables. |
| Page 3, lines 47-48, is it a new sentence for “Living with mother’s only... ”? This part should be checked for typo and reorganized for better understanding. | - Living with mother’s only indicates adolescents/youth were living only with the support of his or her mother. When the data collectors asked them about currently living arrangement of the participants, a numbers of the participant’s responses were living with mother only or father only or living with both mother and father or others options found in the table. |
| **Introduction:** Page 3, line 63, need a citation for the sentence “It is estimated that each year, 200,000 homicides occur in this age group in the world.”. | Thank you,   - Authors accepted the comment and corrected it accordingly. |
| Pages 4-5, lines 82-108, the authors introduced many violence-related studies involving youth around the world. What was the core information the authors tried to deliver? As we all know, the prevalence of violence has been well studied; in some countries, surveillance data are available to monitor the trend and consequences of youth risk behaviors including violence. The authors may want to reorganize the literature and see how they can better serve their paper. | - The core information that the authors tried to deliver was how much the prevalence of youth violence in different countries and settings in general and types of violence in particular. By reviewing different literature, authors identified limited studies on youth violence in Ethiopia, most of the studies were concerned on gender based or intimate partner violence among reproductive age women from 15 to 49 or sexual and reproductive health problems among youth. |
| Page 6, lines 123-129, what are the gaps in the current literature? The authors need to briefly summarize the findings from pervious violence-related studies that included youth in Ethiopia, and then specify the gaps that this study may bridge. If very limited studies were found, studies in other countries with similar demographic characteristics or socio-economic status should be highlighted to provided information instead. | - The gaps in these limited literatures were the studies mainly concentrated among women age 15 to 49 years. On the other hand, analysis from EDHS data that might underestimate the prevalence of violence, other studies were done only on males on a single type of violence or institutional based. Whereas, this study concerned youth violence on both sex groups, community based and also assessed the three forms of violence among youth simultaneously. - Studies in other countries with similar demographic characteristics or socio-economic status might have different socio-cultural and behavioral characteristics. There might be also a variation on the associated factors due to that their results might not represent the results of studies in our country. Therefore, Identifying the prevalence and associated factors of youth violence will be helpful to design prevention and intervention strategies in the study area. |
| **Methods:** Page 7, lines 147-149, the authors mentioned: “Firstly, two woredas from the central Gondar zone, one woreda from each North Gondar zone and west Gondar zone were selected. Secondly, three kebeles were selected from the selected woredas.” How were the woredas and kebeles selected? Details need to be provided. | - This mega project study was done in centeral, west and north Gondar zone. A total 24 woredas present in the three zone: centaral Gondar zone have 13 woredas, north Gondar zone 7 woredas , and west Gondar zone 4 woredas. - “Firstly, two woredas from the central Gondar zone, one woreda from each north Gondar zone and west Gondar zone were selected by using simple random sampling technique. - Secondly, from the total number of kebeles (546 kebeles) in the three zones: three kebeles were selected from each selected woredas by simple randomly sampling. |
| Pages 7-8, lines 154-167, what were the variables/survey questions used in this study? Only definitions from WHO are insufficient for readers to learn this study. The authors may benefit from providing a detailed description of the survey, including how it was developed, included domains/topics, and questions/valid responses used in this study. | Thank You, we have addressed the survey questions in the document.  The survey questions were   - Have you experienced violence in the past 12 months? - Have you experienced violence like hit /slapped or thrown something by someone in the past 12 months? - Have you experienced violence like scared/intimidated by somebody in the past 12 months? - Have you exposed to sexual violence for the last 12 months? |
| Pages 8-9, lines 180-185, was it a complete dataset for analysis? Any missing data information? | - Yes - No any missing data information was found in this study analysis. |
| **Results:** Pages 9-13, table 1 and table 2 can be combined, as both addressed the participants’ demographic information | Thank You,   - We have done it. |
| Page 13, lines 201-205, it seems there were multiple violence variables. Which one was the dependent variable used for the logistic regression? | - The dependent variable used for the logistic regression was “prevalence of youth violence” |
| Page 15, line 201, what were the independent variables that tested in the regression? If all of them were listed in table 4, why they were selected? Are there any other variables in the survey that can be potentially taken into the model (e.g., mental health problems)? | - All independent variables listed in the socio-demographic table and other potential variable from the survey (substance use) were tested in the bi-variable analysis. - Variables listed in table 4(3) (sex, marital status, educational level, currently attending with school, living arrangement, wealth index, and substance use) were selected after bivariable analysis results- variables have a p-value of < 0.2 were taken in to the multivariable logistic regression model. - Potential variable selected in the survey was substance use. |
| **Discussion:** This section repeated too much of the results (e.g., lines 227, 235, 248, 251...). Also, the authors may want to discuss the strengths of this study and implications for violence prevention. | - Thank you. Authors have made correction on the main document. - The strength and implication of this study is included in the main document. |
